# Alcohol and Smoking Affect Risk of Uncomplicated Colonic Diverticulosis in Japan

**DOI:** 10.1371/journal.pone.0081137

**Published:** 2013-12-10

**Authors:** Naoyoshi Nagata, Ryota Niikura, Takuro Shimbo, Yoshihiro Kishida, Katsunori Sekine, Shohei Tanaka, Tomonori Aoki, Kazuhiro Watanabe, Junichi Akiyama, Mikio Yanase, Toshiyuki Itoh, Masashi Mizokami, Naomi Uemura

**Affiliations:** 1 Department of Gastroenterology and Hepatology, National Center for Global Health and Medicine, Tokyo, Japan; 2 Clinical Research and Informatics, International Clinical Research Center Research Institute, National Center for Global Health and Medicine, Tokyo, Japan; 3 Clinical Research Center for Clinical Sciences, National Center for Global Health and Medicine, Tokyo, Japan; 4 Research Center for Hepatitis and Immunology, National Center for Global Health and Medicine, Kohnodai Hospital, Chiba, Japan; 5 Department of Gastroenterology and Hepatology, National Center for Global Health and Medicine, Kohnodai Hospital, Chiba, Japan; University Hospital Llandough, United Kingdom

## Abstract

Colonic diverticula are located predominantly on the right side in Asia and on the left side in Europe and the United States. Factors associated with uncomplicated colonic diverticulosis and its distribution pattern have been unknown. Our aims are to investigate the prevalence and risk factors for uncomplicated colonic diverticulosis. We conducted a prospective cross-sectional study in adults who underwent colonoscopy. Alcohol, alcohol related flushing, smoking, medications, and comorbidities were assessed by interview on the colonoscopy day. Alcohol consumption was categorized as nondrinker, light (1–180 g/week), moderate (181–360 g/week), and heavy (≥361 g/week). Smoking index was defined as the number of cigarettes per day multiplied by the number of smoking years and categorized as nonsmoker, <400, 400–799, and ≥800. A total of 2,164 consecutive patients were enrolled. Overall, 542 patients (25.1%) had uncomplicated colonic diverticulosis located on the right side (50%), bilaterally (29%), and on the left side (21%). Univariate analysis revealed age, male, smoking index, alcohol consumption, aspirin use, anticoagulants use, corticosteroid use, hypertension, and atherosclerotic disease as factors significantly associated with diverticulosis. Alcohol related flushing was not associated with the disease. Multivariate analysis showed increasing age (P<0.01), increasing alcohol consumption (P<0.01) and smoking (P<0.01), and atherosclerotic disease (P<0.01) as significantly associated factors. Alcohol and smoking were associated with right-sided and bilateral diverticula. In conclusion, one in four Japanese adults have colonic diverticulosis (50% right-sided). Age, alcohol consumption, and smoking were found to be significant risk factors for uncomplicated colonic diverticulosis, particularly right-sided and bilateral.

## Introduction

Colonic diverticulosis shows geographic variation in both prevalence and pattern. Diverticulosis is rare in Africa and Asia, but common in the United States, Europe, and Australia [1]–[4]. The anatomic distribution pattern of diverticulosis is predominantly left-sided in the West and right-sided in Asia [1], [4], [5].

In Japan, the prevalence of colonic diverticulosis was 2.1% in the 1960s, but increased to 28% by 1997 [6]–[9]. Recent studies have shown that the current prevalence in Korea is 12% [10]. The increased prevalence of diverticulosis in Asian countries suggests that environmental and lifestyle factors play an important role in its pathogenesis [1], [4], [10].

Because asymptomatic colonic diverticulosis has potential to cause serious complications, such as diverticular bleeding or diverticulitis, it is crucial to understand the true prevalence and risk factors of the disease to prevent associated morbidity and mortality. However, the exact risk factors for uncomplicated colonic diverticulosis other than age remain unknown. Although constipation and low-fiber diet have been widely accepted as etiological factors for uncomplicated diverticulosis [1], [4], [11], [12], a recent study showed that a high bowel movement frequency and a high-fiber diet were associated with a higher prevalence of diverticulosis [13]. Furthermore, a prospective cross-sectional study in Korea and the United States revealed alcohol consumption as a new risk factor for uncomplicated diverticulosis [10], [14]. Therefore, further exploration of diverticulosis risk factors is needed.

The widespread use of colonoscopic examination has enabled increased detection of colonic diverticulosis [10], but few studies have reported on the specific factors associated with diverticulosis, especially in Asia [6], [10]. To investigate the prevalence and to identify possible associated factors or risk factors of diverticulosis, we analyzed comprehensive data obtained from a prospective colonoscopy-based study that collated detailed information on smoking, alcohol consumption, comorbidities, and medications.

## Materials and Methods

### Study design, setting, and participants

We conducted a prospective cross-sectional single-center study in adults who underwent diagnostic colonoscopy between September 2009 and July 2012 at the endoscopy unit of the National Center for Global Health and Medicine (NCGM). The NCGM is an emergency hospital with 900 beds located in metropolitan Tokyo, Japan. The institutional review board at NCGM approved this study (No. 750) and all clinical procedures conformed to Japanese and International ethical guidelines (Declaration of Helsinki). All patients gave informed written consent prior to enrolment. No ethical problems exist with regard to the publication of this manuscript. We used anonymized data from patient medical records.

Inclusion criteria were as follows: (1) >18 years old; (2) Japanese nationality; (3) independence in activities of daily living; (4) able to understand written documents; (5) able to write; (6) asymptomatic patients who needed examination for colorectal cancer due to increasing tumor marker and/or fecal occult blood test results and/or abnormal findings on abdominal ultrasonography, computed tomography (CT), positron emission tomography-computed tomography (PET-CT), magnetic resonance imaging (MRI); or patients who wanted screening for colorectal cancer. Exclusion criteria were as follows: (1) patients who did not provide informed consent; (2) patients in whom total colonoscopy could not be performed; (3) and history of colon resection; (4) acute colonic diverticular bleeding or diverticulitis; (5) severe, continuous, or intermittent gastrointestinal (GI) symptoms such as frequent watery diarrhea and hematochezia within one week of onset to determine appropriate medical treatment. All inclusion and exclusion criteria were fulfilled before patients were enrolled.

### Variables, Data sources, and Measurement

After informed consent was obtained, a detailed questionnaire was completed at the endoscopy unit on the same day as pre-colonoscopy. Patients were asked about their 1) lifestyle habits, 2) medications, and 3) comorbidities in a face-to-face interview with medical staff. For medication history, prescriptions and medical records were reviewed in addition to information provided by the patients to avoid omissions.

Patients were asked the following four questions regarding alcohol consumption: “*Do you drink alcohol*?”, “*What types of alcohol do you usually drink; for example, beer, shochu, sake, wine, gin, vodka, whiskey, tequila, or brandy?*”, “*How many days per week do you drink alcohol?*”, and “*How many glasses of about 180 ml of alcohol do you usually drink per day?*” Then, alcohol consumption was calculated and subjects were categorized as nondrinker, light drinker (1–180 g/week), moderate drinker (181–360 g/week), and heavy drinker (≥361 g/week). Duplicate data were allowed.

The flushing questions consisted of the following two items: “*Do you have a tendency to develop facial flushing immediately after drinking a glass of about 180 ml of beer?*” and “*Did you have a tendency to develop facial flushing immediately after drinking a glass of beer in the first one or two years after you started drinking alcohol?*” For both questions, the choice of answers was “*yes*”, “*no*”, and “*unknown*”. If a subject answered yes to either question, they were considered to be deficient in acetaldehyde dehydrogenase 2 (ALDH2) [15].

The smoking index was evaluated among ever and daily smokers and was defined as the number of cigarettes per day multiplied by the number of smoking years. Then, smoking index was categorized as nonsmoker, <400, 400–799, and ≥800.

Patients were asked about regular use of aspirin, anticoagulants, and oral corticosteroids (prednisolone, methylprednisolone, betamethasone, dexamethasone, or hydrocortisone). The survey form included photographs of these oral drugs, which are approved in Japan. Regular use of medication was defined as oral administration starting at least 1 year before the interview.

Evaluated comorbidities were hypertension, atherosclerotic vascular disease including diabetes mellitus and dyslipidemia, coronary heart disease, and chronic renal failure. Diabetes mellitus, dyslipidemia, and hypertension were considered present in patients taking specific drugs. Chronic renal failure was considered present in patients on hemodialysis or peritoneal dialysis, or with serum creatinine levels ≥2.0 mg/dl.

An electronic high-resolution video endoscope (model CFH260; Olympus Optical, Tokyo, Japan) was used for diagnosis of colonic diverticula. Intestinal lavage for endoscopic examination was performed using 2 L of solution containing polyethylene glycol. If diverticula were observed within the colon, their location type was recorded in the electronic endoscopic database. Distribution type was defined as follows: right-sided, involving the splenic flexure, transverse or proximal colon; left-sided, involving the descending or distal colon; or bilateral, involving the entire colon.

### Statistical analysis

Patients with colonic diverticula were defined as subjects, and those without colonic diverticula were defined as controls, and the relationships between colonic diverticula and clinical factors were examined. To determine risk factors for colonic diverticula, we estimated the odds ratio (OR) and 95% confidence interval (CI). Pearson's Chi-squared test was used to determine the univariate association between each variable and the presence of diverticulosis. In multivariate analysis, we used a multiple logistic regression model. The Cochran–Armitage test was used to identify the trend between each variable and the presence of diverticulosis. A value of P<0.05 was considered significant. All statistical analysis was performed using Stata version 10 software (StataCorp, College Station, Texas, USA).

## Results

### Participants and prevalence

**Figure 1:**
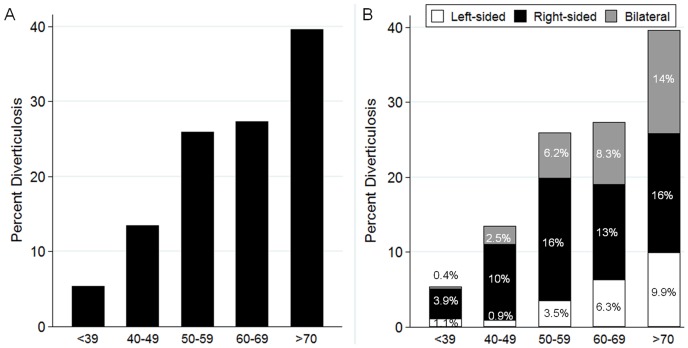
Prevalence of diverticulosis (A) and anatomic distribution (B) by age category (n = 2,164). Colonic diverticulosis increased with age (A). The prevalence of right-sided diverticula was high at for younger age, while left-sided and bilateral types increased with age (B).

During the study period, 2,319 patients participated in medical interviews. Of them, 91 could not undergo total colonoscopy and 64 had a history of colorectal resection. Ultimately, 2,164 consecutive patients comprising 542 patients with uncomplicated colonic diverticula were enrolled. The overall prevalence of colonic diverticulosis was 25.0%. Diverticula were located predominantly in the right-side of the colon in 50.0% (n = 271), bilaterally in 29.3% (n = 159), and in the left-side in 20.7% (n = 112) of cases. The prevalence of colonic diverticulosis increased with age (Figure 1A). The prevalence of right-sided diverticula was significantly higher in younger patients than in older patients (<39 vs 40–59 years, p<0.01; 40–59 vs 50–59 years, p = 0.02), and tended to increase significantly (P<0.01 for trend) with age. Similarly, that for left-sided and bilateral types also tended to increase significantly (P<0.01 for trend) with age (Figure 1B). There were 223 patients (10.3%) who underwent on CT, PET-CT, or MRI. No significant differences in the prevalence of colonic diverticulosis were noted between the group with abnormal imaging findings and the group with normal imaging findings in 23.3% (n = 17/73) and 22.0% (n = 33/150) (P = 0.83) of cases, respectively.

### Risk factors

Table 1 shows patient characteristics. On univariate analysis, age, male, smoking index, alcohol consumption, regular aspirin use, regular anticoagulants use, regular corticosteroid use, hypertension, and atherosclerotic disease were significantly associated with diverticulosis. On multivariate analysis, increasing age, increasing alcohol consumption and smoking, and atherosclerotic disease were significantly associated with diverticulosis (Table 2). Relative risk tended to increase with age, smoking index, and the amount of alcohol consumed (Table 2).

**Table 1:** Characteristics in patients with or without colonic diverticulosis on univariate analysis (n = 2,164).

Variables	All cases (n = 2,164)	With Diverticulosis (n = 542)	Without diverticulosis (n = 1,622)	P
**Mean Age (SD), years**	58 (14)	56 (15)	65 (11)	<0.01
<39	280 (13)	15 (2.8)	265 (16)	
40–49	320 (15)	43 (7.9)	277 (17)	
50–59	374 (17)	97 (18)	277 (17)	
60–69	685 (32)	187 (35)	498 (31)	
>70	505 (23)	200 (37)	305 (19)	<0.01
**Sex (Male)**	1,356 (63)	364 (67)	992 (61)	0.01
**Smoking index** *				
Nonsmoker	1,056 (49)	214 (39)	842 (52)	
<400	533 (25)	93 (17)	440 (27)	
400–799	319 (15)	114 (21)	205 (13)	
>800	256 (12)	121 (22)	135 (8.3)	<0.01
**Alcohol**				
Non-drinker	856 (40)	142 (26)	714 (44)	
Drinker	1,308 (60)	400 (74)	908 (56)	<0.01
**Alcohol consumption**				
Non-drinker	856 (40)	142 (26)	714 (44)	
Light drinker (1–180 g/week)	983 (45)	270 (50)	713 (44)	
Moderate drinker (181–360 g/week)	207 (9.6)	69 (13)	138 (8.5)	
Heavy drinker (≥361 g/week)	118 (5.5)	61 (11)	57 (3.5)	<0.01
**Alcohol Flusher**				
Non-flusher or unknown	1708 (79)	434 (80)	1274 (79)	
Flusher	456 (21)	108 (20)	348 (21)	0.45
**Medication**				
**Regular aspirin use**	206 (9.5)	80 (15)	126 (7.8)	<0.01
**Regular anticoagulants use**	103 (4.8)	40 (7.4)	63 (3.9)	<0.01
**Regular corticosteroid use**	170 (7.9)	26 (4.8)	144 (8.9)	<0.01
**Comorbidity**				
**Hypertension**	745 (34)	264 (49)	481 (30)	<0.01
**Atherosclerotic vascular disease**	644 (30)	223 (41)	421 (26)	<0.01

Categorical variables are reported as n (%).

^*^ The smoking index was evaluated among ever and daily smokers and was defined as the number of cigarettes per day multiplied by the number of smoking years.

**Table 2:** Factors associated with colonic diverticulosis on multivariate analysis.

Variables	Odds ratio (95% CI)	P
**Age**	1.1 (1.0–1.1)	<0.01
**Sex**		
Female	1 (referent)	
Male	1.0 (0.74–1.2)	0.75
**Smoking index** *		
Nonsmoker	1 (referent)	
<400	0.90 (0.66–1.2)	0.47
400–799	1.7 (1.3–2.4)	<0.01
>800	1.8 (1.3–2.5)	<0.01
**Alcohol consumption**		
Non-drinker	1 (referent)	
Light drinker (1–180 g/week)	2.2 (1.7–2.8)	<0.01
Moderate drinker (181–360 g/week)	2.7 (1.8–4.0)	<0.01
Heavy drinker (≥361 g/week)	5.6 (3.6–8.8)	<0.01
**Alcohol Flusher**		
Non-flusher or unknown	1 (referent)	
Flusher	0.87 (0.66–1.1)	0.30
**Medication**		
**Regular aspirin use**		
No	1 (referent)	
Yes	1.1 (0.75–1.5)	0.73
**Regular anticoagulants use**		
No	1 (referent)	
Yes	1.4 (0.88–2.2)	0.16
**Regular corticosteroid use**		
No	1 (referent)	
Yes	0.69 (0.43–1.1)	0.11
**Comorbidity**		
**Hypertension**		
No	1 (referent)	
Yes	1.2 (0.95–1.5)	0.13
**Atherosclerotic vascular disease**		
**No**	1 (referent)	
**Yes**	1.4 (1.1–1.8)	<0.01

^*^ The smoking index was evaluated among ever and daily smokers and was defined as the number of cigarettes per day multiplied by the number of smoking years.

### Distribution type and factors

**Figure 2:**
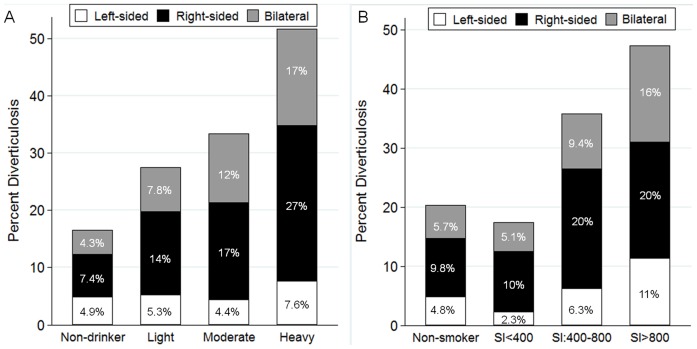
Prevalence of diverticulosis and anatomic distribution by alcohol consumption (A) and smoking index (B) (n = 2,164). Right-sided and bilateral diverticula increased significantly in line with amount of alcohol consumption (A). All distribution types of colonic diverticula increased significantly in line with smoking index (SI) (B).

Right-sided and bilateral diverticula increased significantly in line with alcohol consumption (P<0.01 for trend) (Figure 2A), while left-sided diverticula were not significantly (P = 0.60) associated with alcohol consumption (Figure 2A).

Distribution type of colonic diverticula increased significantly in line with smoking index (left-sided: P<0.01; right-sided: P<0.01; and bilateral diverticula: P<0.01; respectively for trend) (Figure 2B).

## Discussion

In this colonoscopy-based study, we demonstrated that older age and alcohol consumption are strong risk factors for uncomplicated colonic diverticulosis, and the risk increases in line with the amount of alcohol consumed. Furthermore, patients with high pack-years of smoking, hypertension, and atherosclerotic vascular disease were found to be predisposed to colonic diverticulosis. The prevalence of right-sided and bilateral diverticula increased significantly as alcohol consumption and smoking increased.

Several studies have shown that the prevalence of colonic diverticulosis in Japan increased to 28% by 1997 [6]–[9]. Although no available data were available for the 2000s, we found that the prevalence for this period was 25%. To our knowledge, this is the first prospective study to identify the prevalence of colonic diverticulosis in Japan based on colonoscopic findings because most previous studies used barium enema [6]–[9]. Colonoscopy is used worldwide as a standard tool for the detecting colonic cancer and diverticulosis [10], but it can miss diverticula, especially those in the left-sided colon [16]. Thus, the prevalence of diverticula determined in this study is likely to be lower than the actual prevalence.

Age has been found to be an important risk factor for colonic diverticulosis. It has been suggested that patients with diverticular disease have greater rates of collagen cross-linking [17]. In addition, abnormal thickness of muscles of colonic wall, including collagen cross-linking, is promoted by abnormal colonic movement due to a lack of dietary fiber and results in increased intraluminal colonic pressure or fragility of the thickened muscles due to intraluminal pressure changes with age [3], [4].

Consistent with past studies [4], [5], [8], [9], the diverticula in our subjects first developed on the right-sided colon and extended to the left-sided and bilateral colon with aging. Right-sided diverticula in the Japanese has been considered to be of congenital origin [4], thus identification of factors associated with the right-sided type is important to understand the development of colonic diverticula. Why diverticulis is predominantly right-sided in Asian people and rarely so in other populations, who have the same risk factors as those identified in this study, is unclear. It is possible, however, that differences in the sensitivity of the colon to environmental factors are due to variations in characteristics such as the length and muscle thickness of the colon, body weight, and the structure of the neural and humoral systems [4].

In the present study, alcohol intake and amount were not only associated with the entire colonic diverticula but specifically with right-sided diverticula. Song et al. [10] revealed that alcohol drinkers were two times (OR: 2.2) more likely to develop diverticulosis than nondrinkers when assessed by multivariate analysis in a colonoscopy-based study. Sharaha et al. [14] recently conducted a prospective colonoscopy-based study and found that the OR for diverticula was 1.96 with occasional alcohol use and 1.91 in a ≥1 drink per day group as a reference for non-drinkers. Indeed, alcohol intake is likely to have a deleterious effect for the development of diverticula, but no details on the amount of alcohol consumed were available for type of diverticula in their study. As we asked a detailed question with regard to type, times per week, and amount of alcohol, we were able to assess the precise consumption, which is a strength of this study. The biologic mechanisms linking alcohol to diverticulosis are unclear, but may involve colonic motility [18], [19]. Berenson et al. [18] reported that intravenous administration of alcohol consistently decreases recto-sigmoid motor activity and correlates inversely with blood alcohol levels in humans. Wang et al. [19] demonstrated that alcohol inhibits colonic motility mainly through activation of NF-kB, subsequent upregulation of iNOS expression, and the increase of NO release in myenteric plexus in a rat model.

Smoking and the amount of pack-years was also found in this study to be another lifestyle risk factor for colonic diverticula and specifically the right-sided type. Only few data are available on the relationship between uncomplicated colonic diverticula and smoking [10], [13]. Song et al. [10] found that smokers were 30% more likely to develop diverticulosis than nonsmokers after adjustment for important confounders, but this relationship was not statistically significant. Perry et al. [13] assessed smoking history defined as the total number of years smoked and found that patients with diverticulosis had longer tobacco use than those without. Possible mechanisms for the development of diverticulosis may include colonic microflora and colonic motility. Recently, colonic microflora has been shown to play an important role in the development and progression of diverticular disease [20]. Nicotine is known to inhibit the synthesis of proinflammatory cytokines such as interleukin 1 (IL-1) and tumor necrosis factor (TNF) [21], which may alter microflora. Furthermore, previous studies have shown that smoking increases chemical mediators such as vasoactive intestinal polypeptide (VIP) [22] and nitric oxide [23]. Milner et al. [24] revealed that the VIP content of the mucosa and whole wall was increased in diverticular disease. While, Tomita et al [25] reported that the colonic tissue of the diverticular-bearing segments is more strongly innervated by cholinergic nerves than normal segments of the colon. These findings suggest that chemical mediators affect colonic motility and intracolonic pressure, thereby possibly enhancing bulging of the colonic mucosa.

A limitation of this study is that several pathogenic factors reported to be associated with colonic diverticulosis were not included in the analysis; in particular, physical activity, familial and hereditary factors, obesity, and a detailed quantitative dietary history with regard to fiber and fat intake [3], [4], [12]. The absence of these factors could have confounded the relationships between alcohol and smoking. Although we demonstrated that the comorbidity of atherosclerotic vascular disease is associated with colonic diverticula on univariate analysis, this factor is not a true risk factor. We believe that patients with atherosclerotic vascular disease and colonic diverticula have common predisposing factors such as a low-fiber or high-fat diet and low physical activity.

In summary, our study shows that the overall prevalence of colonic diverticulosis was 25%, with 50% of cases on the right side. In addition to age, the amount of alcohol consumption and smoking were found to be identifiable risk factors for the development of uncomplicated colonic diverticulosis. These factors were also associated with right-sided and bilateral diverticula. Patients with atherosclerotic vascular disease are predisposed to colonic diverticula due to similar risk factors. Further study is needed to explore these associations as well as new risk factors from eastern and western countries.
